# Ectopic Salivary Gland Found in the Vocal Cord: A Rare Case

**DOI:** 10.1155/2024/4973164

**Published:** 2024-08-14

**Authors:** Shori Tajima, Niro Tayama, Fumihiko Matsumoto

**Affiliations:** Department of Otorhinolaryngology Juntendo University Faculty of Medicine, Tokyo 113–8421, Japan

## Abstract

**Introduction:**

Ectopic salivary gland is rarely found in the vocal cords; only two cases have been reported in the English literature. To the best of our knowledge, this is the third reported case of this anomaly. *Case Presentation*. A 78-year-old man with hoarseness two years ago visited our department. There were no other symptoms such as cough or sore throat. He had no history of smoking. Laryngeal endoscopic examination revealed a smooth mass in the anterior right vocal cord. We performed surgery under general anesthesia to remove the polyps and improve hoarseness. The histopathological specimen was reported to be salivary gland tissue. We diagnosed ectopic salivary glands of the vocal cord.

**Conclusion:**

It is necessary to consider the possibility of ectopic salivary glands as mass lesions of the vocal cords. Surgical resection is required and long-term follow-up is necessary after surgery.

## 1. Introduction

There are a variety of submucosal mass in the vocal cord such as polyp, cyst, papilloma, amyloidosis, sarcoidosis, granuloma, and malignant tumor. Ectopic salivary glands have been observed in various locations throughout the body, especially near the line connecting the external ear canal and the medial border of the clavicle, but they are rarely found in the vocal cords; only two cases have been reported in the English literature. To the best of our knowledge, this is the third reported case of this anomaly.

## 2. Case Presentation

**Figure 1:**
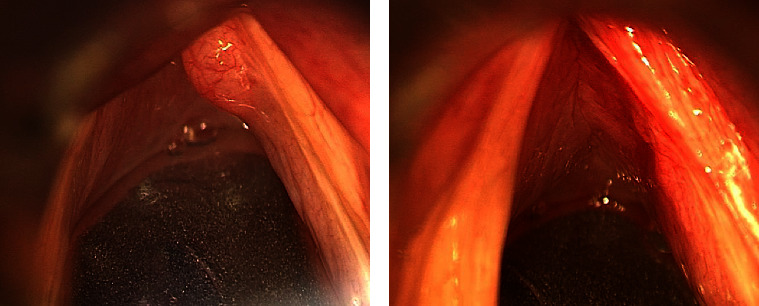
Intraoperative findings through laryngoscopy before and after surgery. (a) Before surgery. A smooth mass in the anterior portion of the right vocal cord. (b) After surgery. Tumor excised, with mucosa preserved.

**Figure 2:**
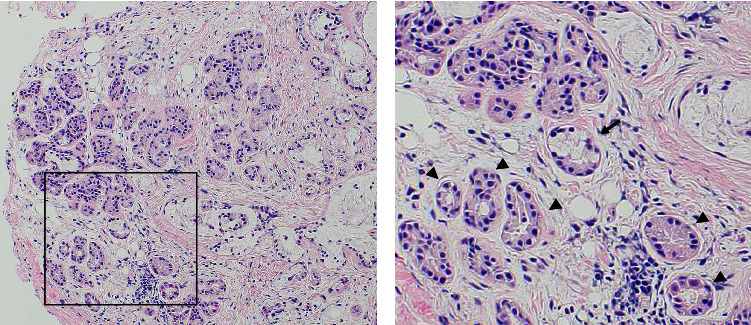
Histological examination. Hematoxylin and eosin stain 200× (a), 400× (b). (a) Histological image of 200× field of view. (b) Histological image of 400× field of view. Serous (arrow heads) and seromucinous (arrow) salivary tissue are scattered.

A 78-year-old man with continuous hoarseness two years ago visited our department. He had history of hypertension and angina pectoris and was taking antiplatelet medication. He had no history of smoking, and his alcohol consumption was moderate. Laryngeal endoscopic examination revealed a smooth mass in the anterior right vocal cord, as shown in Figure 1. Stroboscopy revealed a reduced-wave pattern in the right vocal cord. We performed surgery under general anesthesia to remove the mass lesion and improve hoarseness. The mucosa was preserved and only the submucosal tissue was resected by the microflap technique using cold instruments. We considered laryngeal papilloma as a differential diagnosis; however, the pathological diagnosis during surgery was linear tissue and not a polyp or tumor. The postoperative course was uneventful, and the patient was discharged on the third postoperative day. The histopathological specimen was reported to be salivary gland tissue, as shown in Figure 2. We diagnosed ectopic salivary glands of the vocal cord. No recurrence was observed one year after the surgery. However, careful follow-up is necessary with attention to recurrence. Written approval was obtained from the Institutional Review Board Committee at Juntendo University Hospital Clinical Research and Trial Center (approval number: JHS23-002). Written informed consent was obtained from the patient for the publication of this case report and accompanying images.

## 3. Discussion

An ectopic salivary gland refers to the presence of salivary gland tissue in a location other than where the salivary glands normally exist. It has been reported to occur in all parts of the neck, including the middle ear to the mandible, the thyroid, the pituitary, thyroid gland, cervical lymph nodes, sternoclavicular joints, larynx, and the rectum and vulva. They are especially near the line connecting the external ear canal and the medial border of the clavicle. Ectopic salivary glands are thought to develop by straying during the period from the sixth week to the fourth month of embryonic development; however, it is unclear how salivary gland tissue originating in the oral epithelium migrates to the vocal cord.

To date, only eight cases of ectopic salivary glands of the larynx have been reported in the English literature, as shown in Table 1. Of these, four cases occurred in the false vocal cord [1–3], and only two cases occurred in the vocal cords [4, 5] and laryngeal ventricle [6, 7] each. To our knowledge, this is the third reported case of vocal cord occurrence. The larynx contains mucous lines such as laryngeal glands, but they are distinguished from ectopic salivary glands by the fact that they do not have a luminal structure like salivary glands. Although there is currently no consensus on the optimal treatment strategy for ectopic salivary glands, treatment was based on resection. For ectopic salivary gland, asymptomatic cases do not necessarily require surgery. In this case, surgery was selected because of hoarseness, and postoperative pathology led to the diagnosis of ectopic salivary gland. Ectopic salivary glands do not have a capsule because they are not tumors. It is necessary to resect as much as possible, including the surrounding tissue, with or without the use of a laser because the boundary with the surrounding tissue is not clear. If the salivary gland tissue is left behind, it may recur. Only two cases of recurrence have been reported [3]. All these cases recurred after two or three years, and we believe that long term at least three years follow-up is necessary. Magnetic resonance imaging may help in ruling out a malignant pathology. In conclusion, it is necessary to consider the possibility of ectopic salivary glands as mass lesions of the vocal cords. Surgical resection is required and long-term follow-up is necessary after surgery.

## Figures and Tables

**Table 1:** List of nine cases of ectopic salivary glands of the larynx in the English literature.

Case	Sex	Age	Localization	Treatment	Year	Author
1	Male	56	Laryngeal ventricle	Not mentioned	1999	Kruk-Zagajewska et al.
2	Male	80	Vocal cord	Laser resection	2005	Kempf et al.
3	Male	43	Vocal cord	Surgical resection	2017	Yilmaz et al.
4	Male	46	False vocal cord	Biopsy + follow-up	2018	Tajima et al.
5	Male	56	False vocal cord	Laser resection	2020	Valentino et al.
6	Male	54	Laryngeal ventricle	Surgical resection	2021	Sleurs et al.
7	Female	45	False vocal cord	Surgical resection	2021	Bidaye et al.
8	Male	50	False vocal cord	Biopsy + follow-up	2021	Bidaye et al.
9	Male	78	Vocal cord	Surgical resection	2023	Tajima et al. (present study)

## Data Availability

All data are included within the article.
